# Detecting gas‐induced vasomotor changes via blood oxygenation level‐dependent contrast in healthy breast parenchyma and breast carcinoma

**DOI:** 10.1002/jmri.25177

**Published:** 2016-02-21

**Authors:** Tess E. Wallace, Andrew J. Patterson, Oshaani Abeyakoon, Reem Bedair, Roido Manavaki, Mary A. McLean, James P.B. O'Connor, Martin J. Graves, Fiona J. Gilbert

**Affiliations:** ^1^ Department of RadiologyUniversity of CambridgeCambridge Biomedical CampusCambridgeUK; ^2^ NIHR Cambridge Biomedical Research CentreCambridge Biomedical CampusCambridgeUK; ^3^ Department of RadiologyCambridge University Hospitals NHS Foundation TrustCambridgeUK; ^4^ Cancer Research UK Cambridge InstituteUniversity of Cambridge, Li Ka Shing CentreCambridgeUK; ^5^ Institute of Cancer SciencesUniversity of ManchesterManchesterUK

**Keywords:** BOLD MRI, breast cancer, carbogen, menstrual cycle variation

## Abstract

**Purpose:**

To evaluate blood oxygenation level‐dependent (BOLD) contrast changes in healthy breast parenchyma and breast carcinoma during administration of vasoactive gas stimuli.

**Materials and Methods:**

Magnetic resonance imaging (MRI) was performed at 3T in 19 healthy premenopausal female volunteers using a single‐shot fast spin echo sequence to acquire dynamic *T*_2_‐weighted images. 2% (*n* = 9) and 5% (*n* = 10) carbogen gas mixtures were interleaved with either medical air or oxygen in 2‐minute blocks, for four complete cycles. A 12‐minute medical air breathing period was used to determine background physiological modulation. Pixel‐wise correlation analysis was applied to evaluate response to the stimuli in breast parenchyma and these results were compared to the all‐air control. The relative BOLD effect size was compared between two groups of volunteers scanned in different phases of the menstrual cycle. The optimal stimulus design was evaluated in five breast cancer patients.

**Results:**

Of the four stimulus combinations tested, oxygen vs. 5% carbogen produced a response that was significantly stronger (*P* < 0.05) than air‐only breathing in volunteers. Subjects imaged during the follicular phase of their cycle when estrogen levels typically peak exhibited a significantly smaller BOLD response (*P* = 0.01). Results in malignant tissue were variable, with three out of five lesions exhibiting a diminished response to the gas stimulus.

**Conclusion:**

Oxygen vs. 5% carbogen is the most robust stimulus for inducing BOLD contrast, consistent with the opposing vasomotor effects of these two gases. Measurements may be confounded by background physiological fluctuations and menstrual cycle changes. J. Magn. Reson. Imaging 2016;44:335–345.

Neoangiogenesis gives rise to vessels characterized by structural and functional abnormalities including chaotic vascular networks, caliber variations, increased vascular permeability, and increased interstitial pressure and flow resistance.^1^ The relatively high fraction of immature blood vessels present in solid tumors presents an attractive therapeutic target. However, heterogeneity of vascular maturation has been demonstrated both between and within tumor subtypes.^2^ Histological staining of endothelial cells to alpha smooth muscle actin (α‐SMA) can assess vascular maturation,^3^ but this requires a biopsy, which is invasive, unsuitable for longitudinal measurements, and prone to sampling error. Noninvasive methods that probe vascular function would have potential for characterizing individual tumors, predicting susceptibility to targeted therapies, and monitoring response.

Blood oxygenation level‐dependent (BOLD) magnetic resonance imaging (MRI) is sensitive to changes in the relative vascular deoxyhemoglobin fraction, permitting detection of induced changes in blood oxygenation and flow. BOLD response to various hyperoxic stimulus paradigms has been evaluated in healthy parenchymal organs^4^, ^5^ and in a variety of solid tumors^6^, ^7^ including prostate,^8^ cervical,^9^ head‐and‐neck,^10^ and breast^11^ cancer, to derive potential biomarkers of tumor hypoxia. Administering either pure oxygen or carbogen (conventionally 95% O_2_, 5% CO_2_) increases blood oxygen levels, but with opposite effects on vascular tone, since carbon dioxide is a potent vasodilator. Several groups have therefore proposed exploiting the differential vasomotor effects of hyperoxia and hypercapnia to employ BOLD contrast as a functional biomarker of vascular maturity.^3^, ^12^, ^13^ Immature tumor vessels lacking appropriate smooth muscle vasculature should be unable to either dilate or constrict in response to vasoactive challenges, whereas surrounding healthy mature vessels should show a response.

Magnetic susceptibility effects (the traditional method of BOLD contrast generation) present a particular problem for breast imaging at higher field strengths due to the B_0_ field distortions created by the breast geometry.^14^ Rakow‐Penner et al previously proposed a more robust method to detect BOLD vasomotor contrast in the breast,^15^ deriving BOLD contrast from *T*_2_‐weighted fast spin echo images, rather than *T*_2_*‐weighted gradient‐recalled echo imaging. Oxygen interleaved with carbogen was found to yield the most consistent BOLD response in healthy breast parenchyma compared to air interleaved with either oxygen or carbogen, although the results across subjects were still variable. Immature tumor vessels were expected to exhibit a diminished response relative to surrounding parenchyma, but instead an inverse response to carbogen was observed in two out of three tumor regions they studied, illustrating the complexity of BOLD response in tumors. An optical imaging study measuring hemodynamic response to inspired gas stimuli in the breast has suggested these measurements may be confounded by the body's low‐frequency hemodynamic fluctuations, which may occur due to changes in heart rate, arterial blood pressure, and metabolism.^16^ Furthermore, the spectral amplitudes of background physiological fluctuations in this concurrent study were such that the authors suggested they could be mistakenly attributed to a modulated response to gas stimulus if not properly accounted for.

The aims of this study were to 1) compare the BOLD response to carbogen interleaved with air and oxygen relative to air‐only breathing in volunteers to determine the optimal stimulus design, 2) evaluate the relative efficacy of “carbogen‐light” (2% CO_2_, 98% O_2_) in inducing BOLD contrast, 3) investigate the effect of hormonal variations on response, and 4) evaluate the utility of the optimal stimulus design in a small cohort of breast cancer patients.

## Materials and Methods

### Subjects and Circuit Design

The local Research Ethics Committee approved this study (REC: 14/EE/0145) and all participants gave written informed consent prior to enrollment. Healthy premenopausal females were invited to participate. Volunteers were aged between 22 and 39 years (median age 26 years). The number of days from participants' last menstrual period at the time of the MR examination was recorded as part of a prescreening interview.

**Figure 1:**
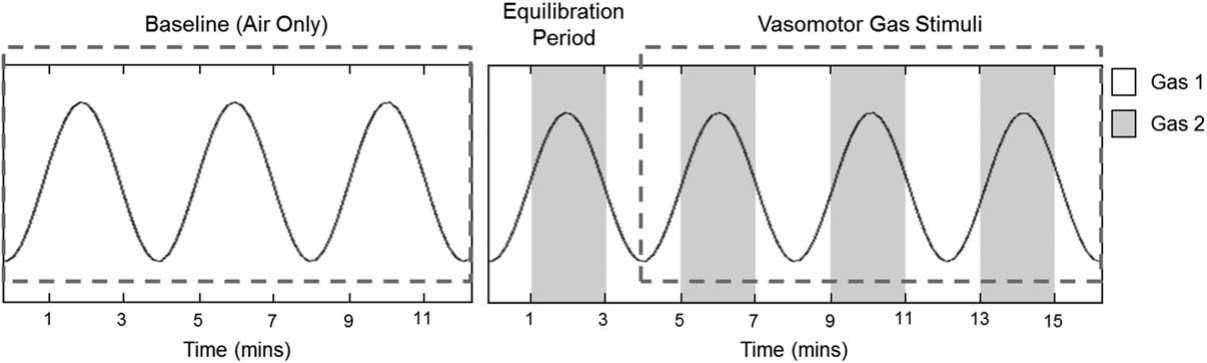
Gas timing diagram for the respiratory stimuli: an initial air‐only control scan was followed by two interleaved stimulus paradigms. The stimulus design alternated between two gases in 2‐minute blocks for a total of 16 minutes; the stimuli variants are detailed in Table 1. The sinusoidal waveform depicts the model used to fit the signal intensity response with a phase offset of zero.

A 12‐minute medical air breathing period was used as a control experiment to determine background modulation in physiology. Subjects then underwent two modulated gas stimulus delivery paradigms with either carbogen or “carbogen‐light” gas mixtures: medical air (21% O_2_) interleaved with carbogen and 100% oxygen interleaved with carbogen. Gases were alternated in 2‐minute blocks for a total of four complete cycles (16 min), as illustrated in Fig. 1. The presentation order of the two modulated gas stimulus delivery paradigms was randomized. The number of volunteers participating in each experiment is listed in Table 1. Gas switching was controlled by an in‐house gas delivery system, comprising a series of valves connected to a digital relay circuit, controlled via MatLab v. 8.3 (MathWorks, Natick, MA). Gases were administered to the subject via an OxyMask (Southmedic, Barrie, ON, Canada)^17^ at a flow rate of 14 L/min, measured by a flowmeter attached to the output of the delivery system. A respiratory band and a finger pulse oximeter were placed on each subject to record respiratory rate, heart rate, and arterial oxygen saturation during the scan. Volunteers were familiarized with the MRI procedure and breathing circuit prior to scanning.

**Table 1:** Number of Volunteers Receiving Each Gas Stimulus Paradigm

	Gas 1	Gas 2	Number of subjects
Stimulus 1	Air	‘Carbogen‐light’	9
Stimulus 2	Oxygen	‘Carbogen‐light’	8
Stimulus 3	Air	Carbogen	9
Stimulus 4	Oxygen	Carbogen	10

### MR Data Acquisition

MRI was performed at 3T (MR750, GE Healthcare, Waukesha, WI) with a dedicated eight‐channel phased‐array breast coil receiver and transmission on the body coil (GE). An in‐house developed multiphase single‐shot fast spin echo (SSFSE) sequence was used to acquire sequential *T*_2_‐weighted sagittal breast images at a single slice location with the following parameters: effective TE 58 msec, TR 4 sec, bandwidth ±83 kHz, matrix size 128 × 128, field of view (FOV) 20 cm, slice thickness 5 mm, and chemical shift selective (CHESS) fat suppression.^18^ A conventional *T*_1_‐weighted image was also acquired at the same sagittal slice location to provide anatomical detail.

### Patient Study

Nine patients aged between 29 and 66 years (median age 51 years) with histologically proven primary breast cancer (tumor diameter >1 cm) who were scheduled to undergo surgery were recruited into this study after providing written informed consent. Patients were scanned using the same BOLD MRI protocol as performed in healthy volunteers, but only the optimal stimulus design was delivered. The sagittal slice was positioned through the maximal tumor diameter, guided by diffusion‐weighted images and *T*_2_‐weighted anatomical images. A dynamic contrast‐enhanced (DCE) protocol was appended to the end of the scan to confirm the tumor site.

### Data Analysis

Several preprocessing steps were applied to the data in order to improve the reliability with which activated regions could be detected. As changes in pixel intensity due to motion can be greater than BOLD activation response, images were registered to minimize noise due to respiratory, cardiac, and involuntary patient motion during the acquisition period. A MatLab implementation of the least squares B‐spline nonrigid registration algorithm described by Rueckert et al was employed to align each image series.^19^ Image registration was also performed to align the baseline and activated scans. A Gaussian filter (full‐width half‐maximum 4 mm) was applied to each image in the series. A straight line was fitted to the signal intensity time course for each pixel and this “trend” line was subtracted from the data in order to eliminate the effect of linear drift. Imaging data from the first alternating gas period were discarded to allow equilibration of the gas inhalation regime.

A region of interest (ROI) was drawn to exclude fat in the outer border of the breast and a pixel‐wise correlation analysis was performed over the visible breast parenchyma in the FOV to explore regions exhibiting a significant response to the alternating stimulus. Following methodology described by Lee et al,^20^ signal intensity response for each pixel was cross‐correlated with a sine and cosine function at the stimulus frequency (0.0042 Hz) representing the 240‐second periodicity of the stimuli. A sinusoidal model was defined to represent the block design stimulus, as the hemodynamic response function effectively acts as a temporal low‐pass filter on the time series.^21^ The cosine function accounts for unknown delays in response. The same frequency was used for the baseline air‐only data, even though there was no imposed periodicity in the stimulus. This yielded the strength of the correlation between background physiological fluctuations and a sinusoid at the stimulus frequency, with a random phase. The magnitude and temporal phase of the correlation are given by the following expressions:

$$r_{m}=\sqrt{r_{s}^{2}+r_{c}^{2}}$$

$$\varphi_{r}={\text{tan}}^{-1}\left(\frac{r_{s}}{r_{c}}\right)$$

where r_s_ and r_c_ are the sine and cosine correlations, r_m_ ranges from 0 to 1 and ψ_r_ ranges from 0 to 2π. Phase lag was defined with respect to Gas 2, ie, phase lag between 0 and π indicates an increase in signal intensity in response to Gas 2 (with some time lag), and a phase lag between π and 2π indicates a signal intensity decrease.

Activation maps were produced showing the correlation coefficient at the phase lag with maximum correlation. Percentage signal deviation from baseline was calculated from an image of the Fourier power at the stimulus frequency and a mean image.^20^ The root mean square (RMS) BOLD signal (y_rms_) in percent was also calculated based on the correlation and measured variance in the time series:

$$y_{rms}=100\times r_{m}\times \left(\frac{\sigma_{y}}{\bar{y}}\right)$$

where σ_y_ is the standard deviation of the time course of each pixel, $\bar{y}$ is the average of the signal, and r_m_ is the correlation coefficient. All image processing and analysis was performed using in‐house programs developed in MatLab v. 8.3.

For the patient analysis, DCE images were reformatted into the sagittal plane and slice‐matched to the BOLD data by correlating morphological features. A radiologist (O.A.) with 4 years experience in breast MRI drew tumor ROIs on slice‐matched postcontrast images at the phase of maximum tumor enhancement, and these were used to delineate the tumor ROIs on the BOLD images. The maximum diameters of the tumor ROIs were 1.63 ± 0.18 cm (range 1.46–1.88 cm).

### Statistical Analysis

All pixels within the fibroglandular tissue ROI that met a certain temporal signal‐to‐noise threshold (TSNR >30) were included in the statistical analysis, rather than selecting a correlation coefficient as a direct threshold for defining activation, to enable an unbiased comparison with the air‐only control state. TSNR was calculated as the ratio of the mean and residual standard deviation of the time course in each pixel, following removal of activation effects by subtracting the scaled model fit from the data. The TSNR threshold was chosen to enable reliable detection of small (∼1%) fluctuations, given the number of timepoints used.^22^ A *P*‐value threshold (*P* < 0.05) was used for the purposes of displaying activation maps and estimating the area of activation. Wilcoxon rank‐sum tests were performed to compare intrasubject correlation distributions, to determine if the alternating stimulus paradigm induced a significant rightward shift of the histogram toward higher correlation coefficients (*P* < 0.001) within fibroglandular tissue relative to the all‐air control.

Summary statistics, including median correlation coefficient, percentage signal deviation, RMS BOLD signal, and area of activation, were calculated for each subject and stimulus. Paired (across subjects) *t*‐tests were performed to compare response to the alternating stimuli and air‐only breathing. The 95th percentile of correlation coefficient was also recorded for each subject's air‐only control state. This provided a measure of the distribution broadness of background physiological noise.

The phase lag at which maximum correlation occurred was evaluated in subjects exhibiting a response to the stimulus that was significantly greater than background physiological variations. Due to the periodic nature of the stimulus, circular mean and standard deviation analysis were used in order to account for phase lag wrapping every 4 minutes (2π) as described previously.^15^

A gas‐to‐air ratio (GAR) metric was calculated as the ratio of median correlation coefficients for the oxygen vs. carbogen stimulus relative to each subject's all‐air control. This gives a measure of the strength of response in healthy volunteers' breast parenchyma due to the gas stimulus relative to each subject's background physiological fluctuations and provides a means of quantitatively comparing the effect of each stimuli.^16^ To evaluate the effect of menstrual cycle changes on BOLD response, this metric was compared between two groups of volunteers scanned in the follicular phase (∼days 10–20), or in the menstrual or luteal phases of their cycle. These groups were chosen as estrogen levels are at their highest between days 10 and 20, taking into account variations in ovulation and cycle length.^23^ A Student's *t*‐test was used to compare the GAR during two different phases of the menstrual cycle. All analyses were performed using R v. 3.1.3 (R Foundation for Statistical Computing, Vienna, Austria).

## Results

Nineteen healthy female volunteers participated in this prospective study. Seventeen volunteers successfully completed the entire imaging protocol. In two subjects, only one interleaved stimulus paradigm plus a baseline air‐only scan was acquired due to a technical issue (failure of fat suppression) and time constraints. Volunteers did not report any adverse side effects as a result of the elevated CO_2_ concentration when breathing 2% or 5% carbogen in 2‐minute blocks interleaved with either medical air or oxygen. No statistically significant change in respiratory (*P* = 0.66, *n* = 9; *P* = 0.12, *n* = 10) or cardiac rate (*P* = 0.15, *n* = 9; *P* = 0.58, *n* = 10) was observed during 5% carbogen breathing compared to air or oxygen breathing, respectively.

### Correlation Analysis

**Figure 2:**
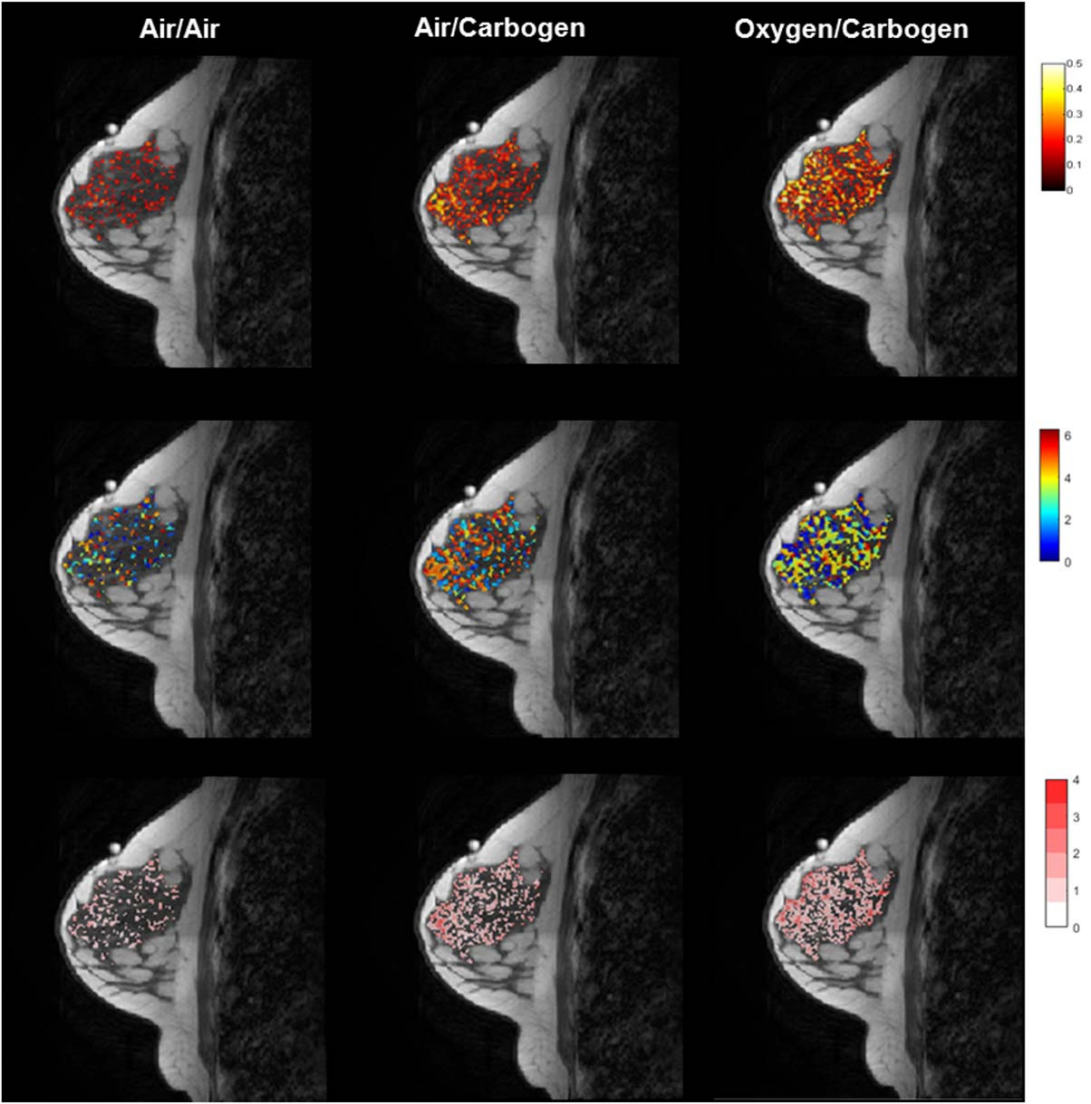
Activation maps (*P* < 0.05) superimposed on a *T*_1_‐weighted anatomy image showing (top row) magnitude of correlation between sinusoidal model and pixel time‐course; (middle row) temporal phase lag (in radians) between sinusoidal model and pixel time‐course; (bottom row) signal deviation (in percent) for the air‐only control, air vs. carbogen and oxygen vs. carbogen in a single volunteer.

**Figure 3:**
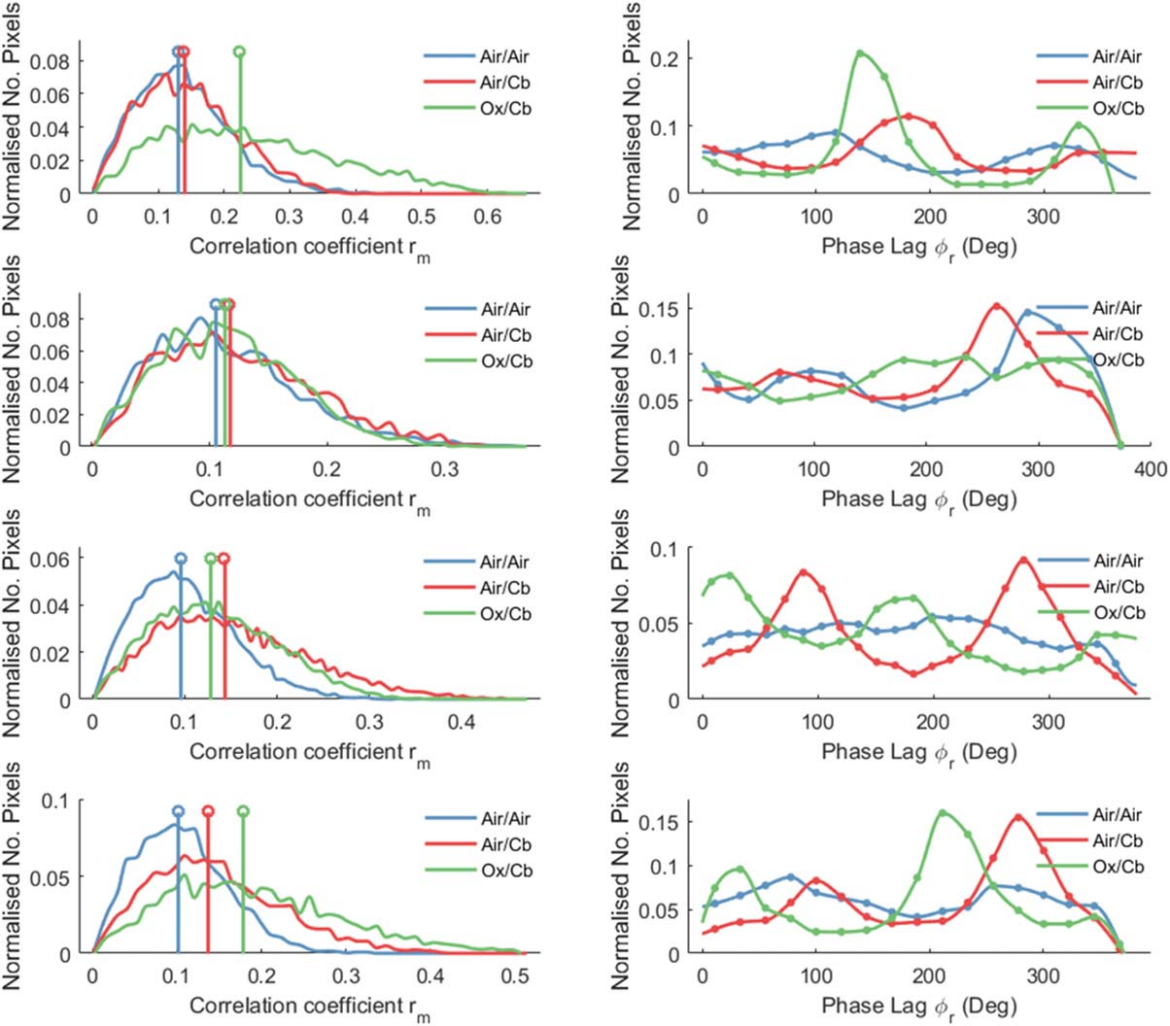
BOLD vasomotor response in four representative volunteers breathing “carbogen‐light” (1–2) and carbogen (3–4) interleaved with air and oxygen, relative to an air‐only control state. Left column: Histograms of correlation coefficients within healthy breast parenchyma. Right column: Histograms showing distribution of phase lags at which maximum correlation occurred.

**Figure 4:**
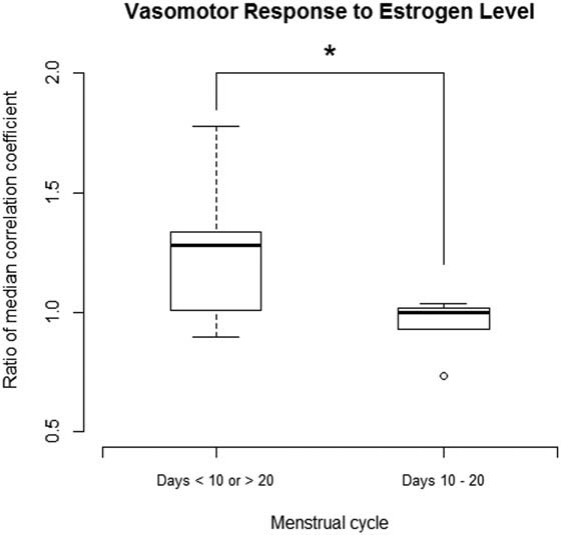
Boxplot showing gas‐to‐air ratio (GAR) of median correlation coefficient for the oxygen vs. carbogen stimulus compared to each subject's all‐air control in healthy breast parenchyma, grouped by phase of menstrual cycle. Subjects scanned during the follicular phase (days 10–20) exhibited a lower response than those scanned in the menstrual and luteal phases (*P* = 0.01), which suggests that higher estrogen levels suppresses vasomotor response.

Figure 2 shows maps of correlation coefficient, temporal phase lag, and percentage signal deviation for the air vs. carbogen and oxygen vs. carbogen stimuli, compared to air‐only breathing for a single volunteer. Histograms of pixel‐wise correlation coefficients (r_m_) for each of the stimulus paradigms are shown in Fig. 3 (left column) for four representative volunteers. A rightward shift in the distribution of correlation coefficients indicates the stimulus paradigm induced an effect that was significantly greater than natural physiological fluctuations. A significant (*P* < 0.001) rightward shift was achieved in 56% of subjects with air vs. “carbogen‐light,” 38% with oxygen vs. “carbogen‐light,” 67% with air vs. carbogen and 70% of subjects who breathed oxygen vs. carbogen. Subjects imaged during days 10–20 of their cycle (follicular phase), when estrogen levels typically peak, exhibited a significantly smaller BOLD response (*P* = 0.01), compared to those in the menstrual or luteal phases of their cycle (Fig. 4).

Table 2 shows the results of paired *t*‐tests comparing the median correlation coefficient, percentage signal deviation, RMS BOLD signal, and area of activation between each stimulus combination and the air‐only control state. Of the four stimulus paradigms tested, oxygen vs. carbogen was the only stimulus to induce a significant shift in median correlation, RMS BOLD signal and activation area compared to the all‐air control. The 95th percentile of r_m_ for the air‐only control state was 0.248 ± 0.064 (coefficient of variation 26%).

**Table 2:** Distributions of Response to Each Stimuli Combination and Statistical Inferences Comparing Each Stimulus to the Baseline Air‐Only State

	Air only Mean ± SD	Air/Cb Mean ± SD (*P‐*value)	Ox/Cb Mean ± SD (*P‐*value)
*‘Carbogen‐light’ (2%), n = 9*			
Median correlation coefficient	0.13 ± 0.03	0.14 ± 0.04	0.15 ± 0.05
	—	(0.19)	(0.08)
Median signal deviation (%)	0.77 ± 0.24	0.90 ± 0.28	0.96 ± 0.38
		(0.16)	(0.13)
Median RMS BOLD signal (%)	0.29 ± 0.08	0.34 ± 0.13	0.34 ± 0.13
	—	(0.15)	(0.14)
Percentage pixels activated (*P* < 0.05)	38.4 ± 15.7	45.8 ± 16.1	47.2 ± 21.2
	—	(0.15)	(0.08)
*Carbogen (5%), n = 10*			
Median correlation coefficient	0.11 ± 0.02	0.13 ± 0.02	0.13 ± 0.02
	—	(0.10)	(0.04^a^)
Median signal deviation (%)	0.60 ± 0.16	0.68 ± 0.15	0.67 ± 0.15
		(0.13)	(0.11)
Median RMS BOLD signal	0.21 ± 0.05	0.24 ± 0.06	0.25 ± 0.06
	—	(0.09)	(0.03^a^)
Percentage pixels activated (*P* < 0.05)	27.9 ± 13.6	39.2 ± 10.0	38.7 ± 12.5
	—	(0.10)	(0.04^a^)

a Significant *P‐*value.

### Temporal Phase Analysis

Figure 3 also shows the distribution of pixels with respect to phase (right column), which represents the temporal lag of each pixel's response. For alternating stimulus paradigms inducing a significant BOLD effect, there exist two clear peaks separated by ∼180°. The second peak represents pixels exhibiting a negative signal intensity response to carbogen, while the first peak represents pixels with a signal intensity increase. In general, the phase response of background variations is more dispersed. Circular means and standard deviations of the phase of maximum correlation are shown in Table 3 for volunteers exhibiting a measurable response to the stimulus paradigm. Most stimulus designs exhibited a circular mean phase lag greater than π radians (2 min), indicating a negative response to Gas 2, ie, the dominant response in parenchyma was a signal intensity decrease in response to carbogen.

**Table 3:** Mean Phase Lags and Standard Deviations Between Gas Stimulus and BOLD Response in Subjects Exhibiting a Significant Response to the Interleaved Stimulus Design Above Background Variations

Gas stimulus	Mean phase lag	Standard deviation	Number of studies
Air/Cb (2%)	2 min 21 sec (1.18π)	28 sec (0.23π)	5
Ox/Cb (2%)	1 min 33 sec (0.78π)	1 min 6 sec (0.55π)	3
Air/Cb (5%)	3 min 26 sec (1.72π)	52 sec (0.43π)	6
Ox/Cb (5%)	3 min 37 sec (1.81π)	42 sec (0.35π)	7

### Patient Study

Eight patients successfully completed the entire imaging protocol. Data were excluded from two subjects where the tumor was incorrectly located prior to intravenous contrast administration. One further dataset was retrospectively excluded due to a lack of parenchyma in the imaging slice for comparison. This left five patients available for analysis. Demographics and tumor characteristics of these patients are shown in Table 4.

**Table 4:** Summary of Five Patient Cases

Patient	Age	Menopausal status	Tumor type	Grade	Receptor status	Tumor size (cm)
1	59	Postmenopausal	Mucinous carcinoma	1	ER+/ HER2‐	2.6
2	52	Postmenopausal	Invasive ductal carcinoma	2	ER+/HER2‐	1.2
3	29	Premenopausal	Invasive ductal carcinoma	2	ER+/HER2‐	1.9
4	51	Postmenopausal	Invasive ductal carcinoma	3	ER+/HER2+	1.5
5	44	Premenopausal	Invasive ductal carcinoma	3	ER+/HER2 ‐	2.3

**Figure 5:**
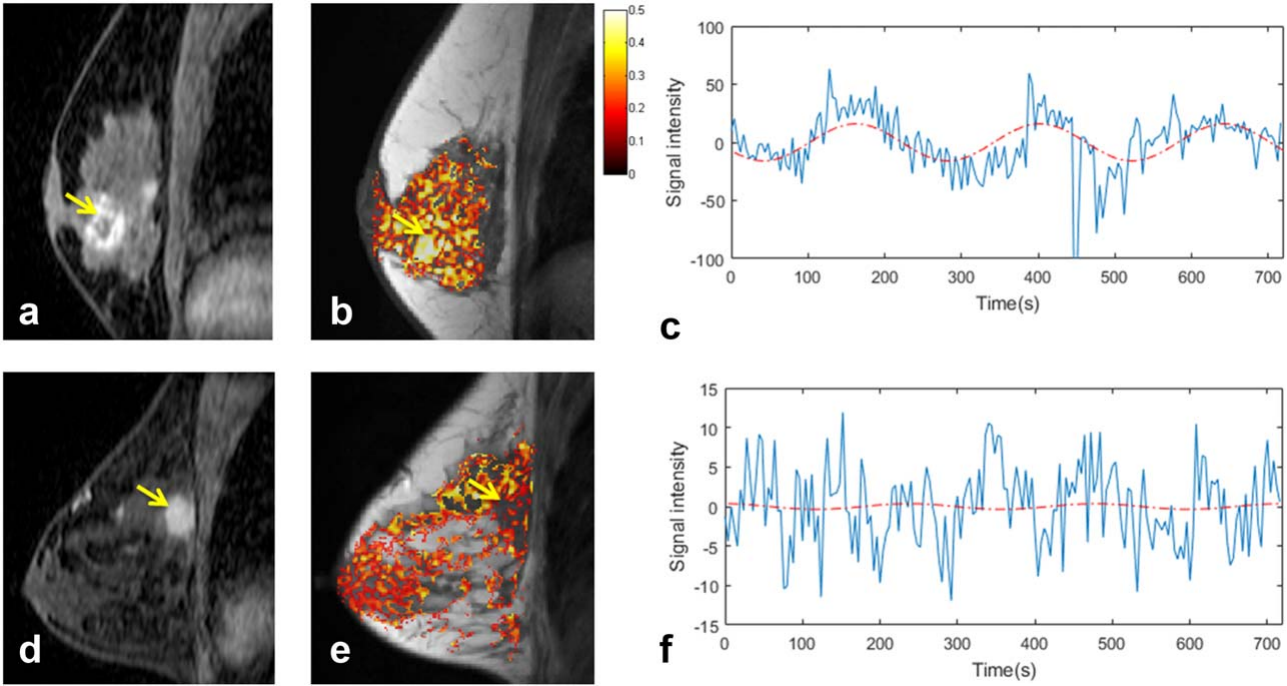
BOLD response to oxygen vs. 5% carbogen stimulus in two patients with (top row) 2.6 cm grade 1 mucinous carcinoma and (bottom row) 2.3 cm grade 3 invasive ductal carcinoma: **a,d:** reformatted sagittal postcontrast enhanced images, yellow arrow denotes tumor; **b,e:** correlation coefficient maps superimposed on *T*_1_‐weighted anatomical image; **c,f:** signal modulation extracted from tumor ROI, with best‐fit sinusoids at the stimulus frequency (phase shifted to match the time lag of response and normalized to the amplitude of response) overlaid in red.

The response in malignant tissue was variable, with two lesions exhibiting a response that was more strongly correlated with the gas stimulus, relative to surrounding parenchyma, while three showed a diminished response, illustrated for two representative patients in Fig. 5. The strength of correlation and time lag of response in tumor tissue and surrounding fibroglandular parenchyma are summarized in Table 5.

**Table 5:** Summary of Correlation Coefficients and Phase Lags Between Gas Stimulus and BOLD Response for Tumor and Surrounding Fibroglandular (FG) Tissue

Patient	Median correlation coefficient		Phase lag at maximum correlation		Phase difference
	FG	Tumor	FG	Tumor	
1	0.23	0.41	2 min 47 sec (1.38π)	40 sec (0.33π)	2 min 27 sec (1.06π)
2	0.21	0.19	13 sec (0.11π)	7 sec (0.06π)	7 sec (0.06π)
3	0.14	0.11	47 sec (0.39π)	47 sec (0.39π)	0 sec (0.00π)
4	0.16	0.25	2 min 40 sec (1.33π)	1 min 53 sec (1.44π)	−13 sec (‐0.11π)
5	0.19	0.11	13 sec (0.11π)	47 sec (0.39π)	−34 sec (‐0.28π)

## Discussion

The aims of this study were to determine which respiratory stimulus design could induce the most robust hemodynamic response in healthy fibroglandular tissue and to explore response in malignant tissue. We evaluated four stimulus paradigms, comprising carbogen and “carbogen‐light” interleaved with either medical air or oxygen relative to an all‐air control state. This investigation builds on previous work to optimize detection of vasomotor response in the breast via *T*_2_‐based BOLD contrast,^15^ as it accounts for normal physiological variations in each subject, which is a confounding factor in these types of analyses.^16^ We also evaluated the efficacy of a lower (2%) CO_2_ carbogen variant for inducing a vasomotor response, as previous studies have reported that subjects found this variant easier to breathe.^24^ The ability of each interleaved stimulus paradigm to modulate hemodynamics was assessed by comparing pixel‐wise correlation distributions for the vasoactive stimuli with subject‐specific air‐only controls. Oxygen vs. carbogen produced a response that was significantly stronger than the all‐air control (*P* < 0.05), with respect to the median correlation coefficient, median RMS BOLD signal, and area of activation. This result supports the theory that BOLD response to carbogen is dominated by changes in vasomotor control, as oxygen vs. carbogen would be expected to induce the largest response due to their opposing vasomodulatory effects.

In clinical patients, three out of the five tumor regions analyzed showed a diminished BOLD response to the oxygen vs. carbogen stimulus relative to surrounding parenchyma. This is an unsurprising result, based on previous histological studies in tumors that reveal inadequate smooth muscle cells lining the vasculature,^3^ which would result in an inability to constrict and dilate in response to vasoactive stimuli. The mucinous carcinoma exhibited a notably large response to the gas stimulus relative to surrounding fibroglandular tissue. Mucinous carcinomas contain large amounts of mucoprotein and therefore exhibit high signal intensity on *T*_2_‐weighted images. They are also characterized by low proliferative activity, leading to slow neovascularization and low oxygen extraction,^25^ which may help account in part for the large changes in blood volume and flow observed in the tumor region in response to the oxygen vs. carbogen stimulus.

Temporal phase lag analysis revealed that phase response in healthy breast parenchyma generally exhibited two peaks, correlating positively and negatively to carbogen, indicating that the mechanism of contrast generation is variable both between and within subjects. The phase spread of each of the groups of data may be attributed to the continuum of transit times of oxygenated blood.^20^ Circular mean phase analysis demonstrated that in most subjects, the dominant response in fibroglandular tissue was a negative correlation with carbogen, ie, signal intensity decreased with carbogen breathing. These results conflict with observations by Rakow‐Penner et al, and with the established brain functional MRI literature, where increased arterial blood flow with carbogen breathing results in a drop in the venous deoxyhemoglobin fraction and a subsequent increase in *T*_2_‐weighted signal intensity. However, studies in normal tissues outside the brain are few, and some of these agree with the current finding of decreased signal intensity with carbogen breathing.^4^, ^5^ This is consistent with a vasodilation‐mediated increase in blood flow and volume, which would increase the amount of deoxygenated hemoglobin in each imaging voxel. With transition to pure oxygen breathing, an increase in signal intensity could arise due to a vasoconstriction‐mediated decrease in blood volume, while maintaining blood oxygen levels. The contrast mechanism for the interleaved air vs. carbogen stimulus is less clear, due to competing effects of elevated blood oxygenation and increased blood volume with carbogen; however, our phase analysis suggests that the vasodilatory effect of carbogen dominates. The degree of vasodilation in healthy tissues is expected to be variable, and may even differ between individuals depending on fitness levels, other metabolic demands, etc. In particular, tissues outside the brain may be less avid and therefore show a steal effect, which could help account for different regions of positive and negative response. We agree with Rakow‐Penner et al that results in patients are highly variable, which is expected due to biodiversity in the underlying tumor vasculature.^15^ A mixed phase response was seen in patients, as three out of the five patients analyzed exhibited a predominantly positive response to carbogen in the fibroglandular tissue region. Additionally, a spectrum of phase differences was observed between healthy and malignant tissue, which may indicate differences in blood delivery, possibly as a result of poor perfusion in tumors.^26^

Analysis of the false‐positive state confirms that low‐frequency hemodynamic fluctuations in normal breast parenchyma may confound measurements of BOLD response to hyperoxic stimuli. Cardiac and respiratory cycles cause large variations at higher frequencies between ∼0.2 and 1 Hz, but due to the temporal resolution of data acquisition (4 sec/image), these may be aliased back into the resulting frequency spectrum. Significant oscillations in blood flow and pressure have also been observed between 0 and 0.4 Hz, with the stimulus frequency 0.0042 Hz overlapping with “very low” frequency variations, attributed to changes in tissue metabolism.^27^ This could be overcome by using the percentile rank of air‐only r_m_ distributions to describe the error probability of assuming pixels above a certain threshold to be “activated.” However, high intersubject variance was observed in the 95th percentile of the air‐only control state data, illustrating the difficulty of defining a single correlation coefficient threshold as a direct indicator of activation. It is interesting to note that the mean correlation coefficient for the oxygen vs. carbogen stimuli in the second cohort is similar to the mean value for air‐only breathing in the first cohort, despite inducing a significant vasomotor response relative to each volunteer's reference air‐only state. Background fluctuations must therefore be accounted for to avoid mistakenly attributing them to the gas stimuli and to allow quantitative comparison between subjects.

Difficulties were observed in inducing a measurable BOLD response in some subjects, with no rightward shift observed in the correlation coefficient histogram, relative to the air‐only state, indicating that in these cases vascular changes were insignificant compared to background physiological noise. Carpenter et al similarly reported that 20% of volunteers breathing air vs. carbogen and 19% breathing oxygen vs. carbogen did not demonstrate a significant effect above natural background fluctuations, likely due to metabolic variations acting independently of the respiratory stimulus.^16^ The ability to detect a significant BOLD response appears to vary with menstrual cycle changes. In general, a larger BOLD response was observed in the menstrual and luteal phases of the cycle when estrogen levels tend to be lower^23^; however, further work is needed to confirm this hypothesis. Differences in breast morphology and composition between subjects may be a further source of intersubject variation in response.

We chose to evaluate both 2% and 5% carbogen gas mixtures in this study, as previous studies have reported that some subjects find carbogen difficult to breathe, leading to high experimental failure rates.^6^, ^7^ Physiological effects of carbogen when breathed for longer periods include a sensation of air hunger and an increased respiratory drive, associated with symptoms of breathlessness and anxiety in some subjects. The original rationale for using 5% carbogen was empirical, but experimental evidence has shown lower CO_2_ concentrations to be much better tolerated by patients, with no compensatory increase in tidal volume or respiratory rate, while still achieving maximum tissue oxygenation.^24^, ^28^ However, no adverse side effects were reported in this study with the 2‐minute block design experiment, with no corresponding increase in cardiac or respiratory rate during carbogen breathing. This stimulus frequency (0.0042 Hz) is typically used in these experiments, as it has a relatively low background physiological noise compared to other frequencies. Greater signal deviation may be seen with a lower stimulus frequency, but at the expense of longer experiment times and reduced subject compliance.

This study demonstrates the utility of oxygen vs. carbogen in inducing a consistent BOLD response with respect to background physiology. The following limitations are noteworthy: due to the prolonged nature of the experiments, two different cohorts were used to compare the carbogen and “carbogen‐light” stimulus paradigms. Variance within subjects is a potential confounding factor in the analysis, although normalizing for background fluctuations should help account for this. In the patient study, an air‐only baseline scan could not be acquired due to time constraints, and so response could not be quantitatively compared between subjects. Furthermore, the small number of patients included in this study means that the results demonstrate feasibility of this technique, but further work is needed to validate it as a biomarker of vascular function.

In conclusion, a measurable BOLD effect was observed in healthy breast parenchyma in response to 5% carbogen interleaved with oxygen, above the demonstrated level of normal physiological variation seen with air. This is consistent with the theory that BOLD contrast is dominated by vasomotor changes. Our results illustrate that control measures of subject physiology are necessary to allow quantitative comparison between subjects. Additionally, estrogen levels appear to confound measurable vasomotor response. The diversity of response seen in tumors suggests this technique warrants further investigation to correlate BOLD contrast changes with histological markers of vessel maturity and therapy response.
